# Genome Sequence of the Fish Pathogen *Flavobacterium columnare* Genomovar II Strain 94-081

**DOI:** 10.1128/genomeA.00430-16

**Published:** 2016-05-26

**Authors:** Salih Kumru, Hasan C. Tekedar, Geoffrey C. Waldbieser, Attila Karsi, Mark L. Lawrence

**Affiliations:** a College of Veterinary Medicine, Mississippi State University, Mississippi State, Mississippi, USA; b United States Department of Agriculture, Agricultural Research Service, Stoneville, Mississippi, USA

## Abstract

*Flavobacterium columnare* causes columnaris disease in fresh and brackish water worldwide. *F. columnare* strain 94-081 was isolated from a diseased channel catfish in 1994; its genome sequence is the first completed genomovar II sequence.

## GENOME ANNOUNCEMENT

*Flavobacterium columnare* is a long Gram-negative rod in the family *Flavobacteriaceae* within the *Bacteroidetes* phylum (1, 2). Several *Flavobacterium* species cause disease in fish, and *F. columnare* is the causative agent of columnaris disease in fresh and brackish water fish (3). *F. columnare* strains are grouped into three major genomovars (4–7), and within the species *F. columnare*, strains vary in their colony morphology and virulence in fish. Our group reported the complete genome sequence of *F. columnare* strain ATCC 49512, which is a representative strain from genomovar I (accession no. CP003222.2) (8). Here, we report the first complete genome sequence of a representative strain from *F. columnare* genomovar II, strain 94-081 (5). Strain 94-081 is virulent in healthy channel catfish (*Ictalurus punctatus*), which is an important aquaculture species in the United States (9).

To obtain the *F. columnare* strain 94-081 genome, a sequencing run was conducted on a PacBio RS II (25× coverage), and *de novo* assembly was conducted using the PBcR pipeline within Whole Genome Assembler version 8.2 beta (10). A mate-pair sequencing run on a MiSeq (Illumina) (5.8 million reads × 75 bp) was conducted, and these reads were mapped onto the PacBio assembly to correct sequence errors, resulting in three contigs >10 kb. Assembly analysis and primer design were conducted using Sequencher 5.3 (Gene Codes Corporation) and CLC Workbench 6.5.1 (CLC bio), and a circular chromosome sequence was obtained by long-range PCR and Sanger sequencing by primer walking.

The final chromosome had 3,321,600 bp, with 30.8% G+C content. For annotation, NCBI Prokaryotic Genome Automatic Annotation Pipeline (PGAAP) and Rapid Annotations using Subsystems Technology (RAST) were used (11, 12). PGAAP annotation showed that the circular *F. columnare* 94-081 genome had 2,779 coding genes, four rRNA operons, and 74 tRNAs. In comparison, the *F. columnare* genomovar I strain ATCC 49512 has 3,162,462 bp, with 31.5% G+C content, 2,632 coding genes, five rRNA operons, and 74 tRNAs.

Function-based comparison of *F. columnare* strain 94-081 and *F. columnare* ATCC 49512 by RAST showed that *F. columnare* strain 94-081 has 34 unique protein-coding genes, including cysteine synthase, teichoic acid export ATP-binding protein TagH, retron-type RNA-directed DNA polymerase, and metal-dependent hydrolases of the β-lactamase superfamily III. *F. columnare* strain 94-081 has 412 unique protein-coding genes (82% are hypothetical); some of these include phage antirepressor protein, type II restriction enzyme BsuBI, and clustered regularly interspaced short palindromic repeat (CRISPR)-associated protein Cas2.

The two-way average nucleotide identity (ANI) between the strain 94-081 and ATCC 49512 genomes was 90.71%, which suggests the two strains being classified in different species (13).

### Nucleotide sequence accession number.

The completed *F. columnare* strain 94-081 genome was submitted to GenBank under the accession no. CP013992.
